# Tracking Invasion Histories in the Sea: Facing Complex Scenarios Using Multilocus Data

**DOI:** 10.1371/journal.pone.0035815

**Published:** 2012-04-24

**Authors:** Marc Rius, Xavier Turon, Víctor Ordóñez, Marta Pascual

**Affiliations:** 1 Department of Evolution and Ecology, University of California Davis, Davis, California, United States of America; 2 Centre d'Estudis Avançats de Blanes, Consejo Superior de Investigaciones Científicas (CEAB-CSIC), Blanes, Spain; 3 Departament de Genètica and Institut de Recerca de la Biodiversitat (IRBio), Universitat de Barcelona, Barcelona, Spain; Northeastern University, United States of America

## Abstract

In recent years, new analytical tools have allowed researchers to extract historical information contained in molecular data, which has fundamentally transformed our understanding of processes ruling biological invasions. However, the use of these new analytical tools has been largely restricted to studies of terrestrial organisms despite the growing recognition that the sea contains ecosystems that are amongst the most heavily affected by biological invasions, and that marine invasion histories are often remarkably complex. Here, we studied the routes of invasion and colonisation histories of an invasive marine invertebrate *Microcosmus squamiger* (Ascidiacea) using microsatellite loci, mitochondrial DNA sequence data and 11 worldwide populations. Discriminant analysis of principal components, clustering methods and approximate Bayesian computation (ABC) methods showed that the most likely source of the introduced populations was a single admixture event that involved populations from two genetically differentiated ancestral regions - the western and eastern coasts of Australia. The ABC analyses revealed that colonisation of the introduced range of *M. squamiger* consisted of a series of non-independent introductions along the coastlines of Africa, North America and Europe. Furthermore, we inferred that the sequence of colonisation across continents was in line with historical taxonomic records - first the Mediterranean Sea and South Africa from an unsampled ancestral population, followed by sequential introductions in California and, more recently, the NE Atlantic Ocean. We revealed the most likely invasion history for world populations of *M. squamiger*, which is broadly characterized by the presence of multiple ancestral sources and non-independent introductions within the introduced range. The results presented here illustrate the complexity of marine invasion routes and identify a cause-effect relationship between human-mediated transport and the success of widespread marine non-indigenous species, which benefit from stepping-stone invasions and admixture processes involving different sources for the spread and expansion of their range.

## Introduction

Selective forces and demographic processes have shaped community composition and biogeographic patterns of the world's ecosystems over millions of years of evolution [1]. Genetic tools have enabled researchers to infer the evolutionary history and to understand the biogeography of many taxa in order to reveal how these processes have occurred [2]–[4]. However, the recent increase in large-scale environmental impacts of anthropogenic origin [5] has extensively modified evolutionary trajectories of populations across species ranges [6]. Biological invasions are a direct consequence of such broad human-driven habitat alteration, constituting a crucial factor shaping biodiversity and biogeographic patterns worldwide [7]. The recent increase in research on biological invasions has stimulated debates regarding the evolutionary importance of such invasions [8], [9] and the key aspects of the invasion process such as its long-term consequences and predictability [10]–[12].

Genetic studies have been recognized as crucial to uncover the pathways of the introduction of non-indigenous species (NIS), colonisation histories, and the origin of these introductions [13], [14]. However, genetic studies of NIS have certain methodological, and especially analytical, limitations [15], [16]. For instance, assignment tests and dendrograms might fail to provide accurate assignments of introduced populations, especially when studies analyse populations that have been under severe genetic drift during and after introduction, or that have multiple or unsampled sources [17]. New analytical tools, such as the approximate Bayesian computation (ABC) methods [18]–[21], have recently been utilized in studies of biological invasions to overcome some of these limitations and reconstruct demographic history using genetic data [22]–[26].

Most research studies which have successfully implemented ABC methods have focussed on terrestrial ecosystems, analysing species in their native environment [27], [28] and assessing historical processes within their introduced range (e.g. [22], [23], [26], [29]). In marine ecosystems only a few studies have looked at long-term evolutionary scenarios using ABC methods (e.g. [30], [31]), while no study to date has used this approach to investigate marine NIS. The use of ABC methods in studies of marine NIS is particularly relevant because the sea contains ecosystems that are amongst the most heavily affected by biological invasions worldwide [32]. Furthermore, there is a growing recognition that marine biological invasions are often extremely complex due to the high prevalence of multiple sources and non-independent introductions [15], [33].

The majority of the species responsible for marine invasions are from lower trophic levels [34]. Among these, sessile filter feeder invertebrates are recognized as one of the most important groups [35], [36]. Most of these organisms have a planktonic larval stage with limited dispersal capabilities [37]–[39]. However, larvae can be caught in ballast pumps and survive in transit to other harbours, or adults attached to floating structures such as drift algae or loose debris can be pumped in or gravitated into the ship (through large openings where the water simply flows into the vessel) [40]. In addition, adults can also be transported as fouling on the hulls and sea chests of ships and recreational vessels [41]–[43], and can release their offspring in the locations where these ships stop.

Here, we studied a sessile marine invertebrate, *Microcosmus squamiger* (Tunicata, Ascidiacea), to infer the colonisation histories and routes of invasion of its world populations. We obtained a dataset comprising microsatellite and mitochondrial DNA (mtDNA) data from populations located within the vast distributional range of *M. squamiger*, and analysed them using a variety of analytical tools, including the ABC methods. We specifically compared different scenarios to understand: (1) whether the introductions were independent or not, (2) the origin of the colonisers, and (3) the sequence of the different introduction events worldwide.

## Methods

### Studied species

The solitary ascidian *Microcosmus squamiger* is native to Australia [33], [44], [45] but is now well established on most continents. This species was first recorded outside its introduced range in Bizerte (Tunisia) in the early 1960s [46], but has since been recorded all over the western Mediterranean Sea and adjacent Atlantic Ocean region [47], [48]. Subsequently, this species was detected in California in 1986 [49], on the NE Atlantic coast in 1994 [50], [51], in South Africa in 2000 [52], in New Zealand in 2003 [53], in India in 2006 [54], and recently in Japan (T. Nishikawa, pers. comm.). However, the first report of *M. squamiger* outside its native range might in fact have been from along the southern African coast. Millar [55], [56] reportedly found the congeneric *Microcosmus exasperatus* in surveys conducted between 1950 and 1956 along this coastline. However, considering that: 1) Both *M. squamiger* and *M. exasperatus* are widespread species that can easily be confused [47], 2) Millar's descriptions [55], [56] were oversimplified and not sufficiently detailed to distinguish between the two species, and 3) *M. exasperatus* has never again been reported along the coast of South Africa [52], [57]; it is highly likely that Millar [55], [56] misidentified the samples and that they were in fact *M. squamiger* (see also [52]). Consequently, we consider that the first records of *M. squamiger* as a NIS could have been in South Africa in the 50 s and in the Mediterranean Sea in the 60 s, and not in 1983 as stated elsewhere [58]. Within the localities of its introduced range, *M. squamiger* is abundantly found on both natural and artificial substrata [59]–[61]. It can generally be found in or close to large shipping harbours or marinas [47], [61]–[63], and has occasionally been found in open coastal habitats where *M. squamiger* can be highly invasive and colonise all available hard substrata forming dense aggregates [47], [59]. Ascidians are sessile organisms that have very poor dispersal capabilities, restricted to their short-lived lecithotrophic larvae [37], [38]. Consequently, the natural active spread of *M. squamiger* is restricted to an extremely limited swimming period [64]. Thus, transoceanic relocation of this species is through shipping [33], [47], which makes tracking the movement of NIS very challenging [65], [66].

### Sample collection

**Figure 1:**
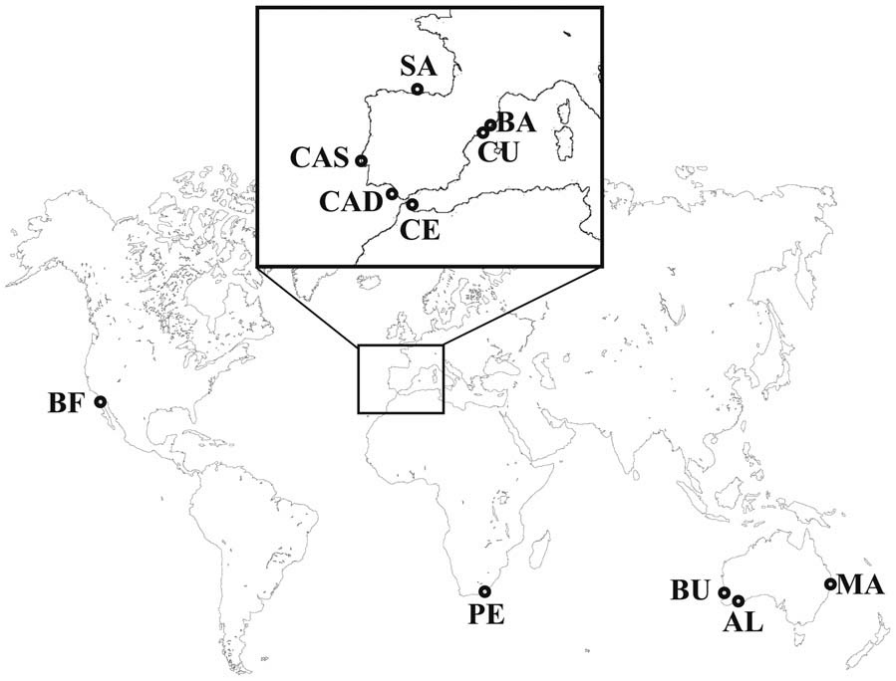
Map of the sampled sites of *Microcosmus squamiger*. The Atlanto-Mediterranean region has been enlarged. Collection sites are abbreviated as in Table 1.

We sampled *Microcosmus squamiger* specimens from 11 sites covering most of the introduced and native ranges of the species (see Table 1 and Fig. 1). No specific permits were required for the described field sites. We collected 24–28 specimens per sampling site through SCUBA diving or by pulling up harbour ropes, ensuring in all cases a distance of a few meters among the different individuals when collected. We dissected the specimens *in situ* and a piece of muscular tissue from the mantle was immediately preserved in absolute ethanol. Once in the laboratory we replaced the ethanol with new absolute ethanol and stored the samples at −80°C until DNA extraction.

**Table 1:** Collection sites of *Microcosmus squamiger* including geographical regions, population abbreviations (Code), type of habitat (O: outside harbour, I: inside harbour) and number of individuals analysed.

	Geographical region	Country	Sites	Code	Latitude/Longitude	Habitat	Sample size	He	H_O_	Na	Np	AR	F_IS_	*h*
Native populations														
	Australasia	Australia	Bunbury	BU	33°19′13″S/115°39′39″E	I	24	0.558	0.514	5.17	1	4.935	0.081	0.692
	Australasia	Australia	Albany	AL	35°01′56″S/117°53′25″E	O	24	0.611	0.512	5.17	4	4.965	**0.165**	0.712
	Australasia	Australia	Manly	MA	27°27′10″S/153°11′22″E	O	24	0.535	0.410	5.50	8	5.146	**0.238**	0.867
Introduced populations														
	North America	Mexico	Bahía Falsa	BF	37°56′18″N/122°25′36″W	O	24	0.550	0.431	4.67	1	4.504	**0.220**	0.825
	Southern Africa	South Africa	Port Elizabeth	PE	33°57′60″S/25°38′06″E	I	24	0.531	0.438	4.17	1	4.029	**0.179**	0.596
	NE Atlantic Ocean	Spain	Santander	SA	43°27′45″N/3°47′22″W	I	28	0.622	0.548	5.17	0	4.900	0.121	0.641
	NE Atlantic Ocean	Portugal	Cascais	CAS	38°41′34″N/9°25′03″W	I	24	0.529	0.354	4.50	0	4.366	**0.335**	0.841
	NE Atlantic Ocean	Spain	Cádiz	CAD	36°31′51″N/6°17′03″W	I	24	0.515	0.394	4.00	0	3.811	**0.239**	0.674
	Mediterranean Sea	Spain	Ceuta	CE	35°53′43″N/5°18′44″W	I	24	0.546	0.511	4.83	4	4.691	0.065	0.587
	Mediterranean Sea	Spain	Cubelles	CU	41°11′37″N/1°39′17″E	O	24	0.545	0.445	4.50	2	4.449	**0.187**	0.853
	Mediterranean Sea	Spain	Barcelona	BA	41°20′33″N/2°09′41″E	I	24	0.509	0.444	4.50	1	4.311	0.129	0.875

Diversity estimates based on microsatellites are as follows: He - mean expected heterozygosity (Nei's gene diversity), H_O_ - mean observed heterozygosity, Na - mean number of alleles per locus, Np - number of private alleles, AR - mean Allelic Richness, F_IS_ - inbreeding coefficient with significant values in **bold**, and. *h* - haplotype diversity in COI (data from [33]).

### DNA extraction, PCR amplification and genotyping

We extracted DNA from each individual using the REALPURE extraction kit (Durviz, València, Spain). We amplified by PCR six polymorphic loci (MS6, MS7, MS10, MS11, MS12 and MS13) that had been isolated for this species [67]. The PCR conditions used were based on 20 µL total reaction volume, with 0.5 µL of each primer (10 µM), 2.5 µL dNTPs (10 mM), 4 µL 5× buffer, 1.8–3 µL MgCl_2_ (25 mM), 9.5 - 8.3 µL H_2_O, 1 U Taq polymerase (Promega) and 1 µL DNA. An initial denaturation at 94°C for 5 min was followed by 30 cycles consisting of a denaturation step at 94°C for 1 min, an annealing step at 53 to 57°C (see [67] for details) for 30 sec and an extension step at 72°C for 30 sec, and a final extension at 72°C for 5 min. The forward primer of each locus was labelled with different fluorescent dyes [67]. We estimated allele sizes based on the standard Rox (70–500 bp, Bioventures) using a capillary sequencer 3730 DNA Analyzer (Applied Biosystems) and the software GeneMapper™ (v. 3.5, Applied Biosystems) from the Serveis Científico-Tècnics of the Universitat de Barcelona. In addition to the microsatellite genotypes, we obtained mtDNA sequences of the same individuals from a previous study [33].

### Analysis of genetic diversity and population structure using microsatellite data

We used the GenAlex programme v. 6.1 [68] to calculate allele frequencies found in each population and microsatellite locus and to run a Mantel test to compare pairwise-population matrices, and to convert our data file to required formats for other programmes. We calculated linkage disequilibrium between pairs of loci in each population using Genepop v. 4.0 [69], while we used Genetix v. 4.05.2 [70] to test deviations from Hardy-Weinberg equilibrium using the inbreeding coefficient (F_IS_) and estimated its significance (10000 permutations).

We assessed pairwise population differentiation using the microsatellite dataset and the *D* estimator [71] computed with the programme DEMEtics v. 0.8–3 [72]. The significance of the pairwise comparisons was evaluated using the built-in randomization procedure of DEMEtics. The use of *D* has been advocated to overcome some of the shortcomings of conventional statistics such as F_ST_ or G_ST_, as it performs better when the goal is to estimate genetic differentiation [72], [73]. Nonetheless, and following recent advice [73], we also estimated population divergence using the conventional F_ST_ estimates for comparison, while a randomization test was used to test the existence, or lack thereof, of significant genetic differentiation for each population pair across all loci. These analyses were done using the Arlequin v. 3.5 programme [74]. For both measures of population differentiation we corrected pairwise *P*-values using the sequential Bonferroni method [75].

### Identification of clusters of genetically related individuals and spatial ordination of between-group structures

In order to have a visual assessment of between-population differentiation, we performed a discriminant analysis of principal components (DAPC) [76]. This technique extracts information from genetic datasets (multivariate in nature) by first performing a principal component analysis (PCA) on pre-defined groups or populations, and then using the PCA factors as variables for a discriminant analysis (DA), which seeks to maximize the inter-group component of variation. The previous PCA step ensures that the variables input to DA are uncorrelated [76].

We performed DAPC using the *adegenet* package for R [77]. A file including microsatellite and mtDNA data was used and DAPC was performed (function dapc) using pre-defined groups corresponding to populations or groups of populations (see Results). Variables were centred but not scaled. In all analyses 50 principal components of PCA were retained as input to DA. The procedure also provides estimates of the probability with which the DA recovers the true group membership of the individuals.

The programme STRUCTURE v. 2.3 was used to detect the number (K) of genetically homogeneous populations in our microsatellite dataset [78]. We used the Admixture and loc prior model because it performs better than other models for detecting genetic structure even in situations of low levels of genetic divergence or a limited number of loci [79]. Following the recommendations of Evanno et al. [80], we calculated an ad hoc statistic IncK based on the rate of change in the log probability of data between successive K-values, since it provides a good estimator to accurately detect the number of population groups. For each dataset we quantified, using 20 runs, the mean and standard deviation of the likelihood of each K. We tested a range of K values depending on the number of populations included in the analysis: 1 to 13 (when all populations were included), 1 to 5 (for native populations) and 1 to 10 (for introduced populations). CLUMPP v.1.1.2 [81] was used to merge the results across the 20 runs for the best selected K while DISTRUCT v.1.1 [82] was used to visualize the results.

### Unravelling the routes of invasions and colonisation histories using ABC methods

In order to obtain relevant and detailed information of the routes of invasions and colonisation histories of *M. squamiger*, we designed a series of sets of evolutionary scenarios and analysed them with ABC methods using the DIYABC v. 1.0.4.41 programme [83]. Given that mtDNA could be affected by adaptive selection [84], we first ran all analyses using microsatellites and subsequently used a combined dataset of microsatellites and mtDNA. Preliminary simulations indicated an absence of genetic bottlenecks when comparing introduced and ancestral populations, and no founder effects in introduced populations (see Results). Thus, we did not consider the presence of bottlenecks for the different sets of competing scenarios. In some scenarios we incorporated the presence of an unsampled population, which acted as an invasive bridgehead population (see [26]), from which other introductions originated.

In order to define each set of scenarios we used prior distributions of demographic parameters (Table S1) based on information regarding the biological and invasion traits of the studied species. To estimate the time of events (in number of generations) for this species, we considered that *M. squamiger* has two overlapping generations per year based on what is know about its life cycle [59]. To estimate the effective population size of each population, we considered information on past geological processes of the Australian region and marine vectors of invasive species. During the late Pleistocene, periglacial deposits formed as a result of cold-climate processes in Australia [85], which most-likely prevented *M. squamiger* from surviving in this area. At the beginning of the Holocene, the global climate became warmer [86] and Australia acquired a climate similar to that found today, allowing species such as *M. squamiger* to thrive in this area. Consequently, we considered that the populations included in this study could have originated ca. 10000 years BP (Table S1). Regarding the introduced populations, we assumed that all *M. squamiger* introductions occurred after the first European sailors visited the Australian shores around 400 years BP [87] (Table S1). Regarding extra-range colonisations, taxonomic records indicate that *M. squamiger* has been outside Australia for at least 60 years, and thus we assumed that introductions within the introduced range only occurred over the last 100 years. Finally, we assumed that the effective population size was the same for all populations and used a uniform distribution bounded between 10–100000 individuals (Table S1).

The first set of scenarios aimed to infer whether the colonisations within the introduced range were independent or not. For this we first divided the introduced populations into four groups according to major geographical regions as defined in Table 1. Three scenarios were compared: two that considered independent colonisations from Australia to the different regions of the introduced range, and one that incorporated an unsampled population from where all introduced populations originated as a result of a non-independent colonisation (see Fig. S1A). In order to establish the sequence of colonisation for the two independent scenarios, we used the taxonomic records of each region as a guideline, and because the Mediterranean Sea and South Africa were colonised around the same time, we interchanged the order of appearance of these regions in the two independent scenarios.

The second set of scenarios focussed on the origin of the introduced populations. For this, we first considered each of the native populations as separate entities, and then followed the outcome of the analysis of the previous set of scenarios (see Results) to group the introduced populations together. Regarding the ancestral populations, both the DAPC and STRUCTURE (see below) showed that Bunbury and Manly were the closest populations genetically to the introduced cluster, while Albany remained separated. Accordingly, preliminary simulations revealed that the exclusion of the population of Albany from the analyses did not affect the final outcome. We thus excluded this population from subsequent analyses. In the first scenario we considered that the introduced populations originated from Manly, as suggested by mtDNA data [33]. The second scenario considered that the colonisers originated from Bunbury. Finally, a third scenario contemplated an admixture event that involved these two Australian populations (Fig. S1B).

The third set of scenarios aimed to assess the sequence of worldwide colonisations across regions. We first grouped the populations according to geographic regions as in Table 1 and assumed that all introduced populations originated from an unsampled ancestral population (see Results). As stated above, the faunistic records indicate that the first introduction of *M. squamiger* was in South Africa or the Mediterranean Sea, followed by California and the NE Atlantic. However, because recurrent colonisations might have obscured the reconstruction of the introduction history of this species, we did not limit our analyses to what the faunistic records indicated but included several other geographical sequences (see Fig. S1C). This approach allowed us to simultaneously examine the independence of the different introductions from an ancestral bridgehead population and the colonisation sequence that these introductions followed.

For all sets of scenarios we used 10^6^ simulated data per scenario to build a reference table. To compute posterior probabilities of the competing scenarios, we used the 1% of the simulated datasets closest to the observed data (using Euclidean distances between each simulated and observed dataset) to estimate the relative posterior probability (with 95% confidence intervals) of each scenario with a logistic regression [19]. For each set of scenarios, the most likely scenario was the one with the highest posterior probability value and non-overlapping 95% confidence intervals, using both a dataset with only microsatellite loci and one that combined microsatellites and mtDNA.

We assessed the sensitivity of different priors that included different effective population sizes and mutation models for the combined dataset of microsatellites and mtDNA. For this, we performed simulations using the first set of scenarios, which focussed on whether or not the colonisations within the introduced range were independent. Different prior sets were used to test the robustness of demographic estimates as follows: Prior set 1) standard priors as described in Table S1; Prior set 2) uniform distributions of effective population sizes bound between 10 and 10^6^ diploid individuals for N, Nau and Nu; Prior set 3) stepwise mutation model for microsatellite loci; and Prior set 4) no insertion-deletion mutation rate in microsatellites flanking regions.

## Results

### Genetic diversity and differentiation of *M. squamiger* populations

A total of 268 individuals were genotyped using six microsatellite loci. The number of alleles per locus ranged from 4 at the MS7 locus to 18 for the MS10 locus (Table S2). No linkage disequilibrium was observed between loci pairs after correction for multiple tests by false discovery rate [88] and thus all loci were considered independent. The mean expected heterozygosities were higher than the observed ones in all populations and the global F_IS_ values were significant in all cases except for Bunbury (Australia) and three sites in Spain (Santander, Ceuta and Barcelona) (Table 1). The significant homozygote excess was mainly due to loci MS11 and MS12 in the remaining introduced populations (Table S3). The population that showed the highest number of private alleles was Manly, Australia (Table 1). On average, native populations showed higher numbers of private alleles than the introduced ones (mean ± S.E., 4.33±2.03 and 1.12±0.48, respectively) but the differences were not significant (*t*-test, *t* = −1.54, *P* = 0.25). The mean allelic richness was significantly higher in native than in introduced populations (mean ± S.E., 5.01±0.07 and 4.38±0.12, respectively; *t*-test, *t* = −4.55, *P*<0.01). However, when we analysed the numbers of mtDNA COI haplotypes [33], no significant differences among native and introduced populations were found (mean ± S.E., 10±1 and 7.87±0.85 respectively; *t*-test, *t* = −1.62, *P* = 0.16). Both native and introduced populations showed similar microsatellite heterozygosity (mean ± S.E., 0.568±0.02 and 0.54±0.01, respectively; *t*-test, *t* = −0.96, *P* = 0.40) and mtDNA haplotype diversity (mean ± S.E., 0.757±0.05 and 0.736±0.04, respectively; *t*-test, *t* = −0.29, *P* = 0.78). When we compared the observed and expected gene diversity of the microsatellite alleles found in each population, the programme BOTTLENECK [89] did not detect bottlenecks for the populations of the introduced range (Table S4). We used IAM and TPM mutation models given the allele size distribution found in the *M. squamiger* microsatellite dataset.

The mean *D* in pairwise comparisons was smaller among introduced (0.005±0.006, mean ± SE) than among native (0.227±0.058) populations, and the same was found for the F_ST_ estimates (0.047±0.006 and 0.105±0.027, respectively). After correcting for multiple tests, 38 out of 55 *D* values indicated significant genetic differentiation (Table 2), while 17 comparisons were not significant, of which 16 involved introduced populations. Pairwise F_ST_ values yielded very similar results (Table 2), although in this case 22 comparisons were not significant, again involving mostly (20 pairs) introduced populations. Both estimators of population-pairwise differentiation were strongly correlated as shown by the Mantel test (r = 0.922, P<0.001).

**Table 2:** Measures of genetic differentiation based on the microsatellite loci in pairwise comparisons of the studied populations of *Microcosmus squamiger*.

	BU	AL	MA	BF	PE	SA	CAS	CAD	CE	CU	BA
**BU**		**0.1303**	**0.2209**	**0.0648**	**0.1054**	**0.1504**	0.0532	**0.1227**	**0.1164**	**0.1327**	**0.1040**
**AL**	**0.0519**		**0.3304**	**0.2497**	**0.2834**	**0.3468**	**0.2622**	**0.3017**	**0.2850**	**0.3399**	**0.2843**
**MA**	**0.1261**	**0.1374**		**0.1507**	**0.1227**	**0.2346**	**0.1321**	**0.1853**	**0.1174**	**0.1661**	**0.1341**
**BF**	0.0324	**0.0860**	**0.0941**		0.0323	**0.0707**	0.0404	**0.0752**	0.0182	0.0401	0.0342
**PE**	**0.0581**	**0.1081**	**0.0899**	0.0188		**0.1199**	0.0215	**0.0538**	0.0344	**0.0557**	0.0338
**SA**	**0.1087**	**0.1351**	**0.1370**	**0.0624**	**0.0864**		**0.1090**	**0.1089**	**0.0732**	0.0341	**0.0780**
**CAS**	0.0258	**0.1067**	**0.0945**	0.0227	0.0128	**0.0766**		0.0061	**0.0569**	0.0323	0.0189
**CAD**	**0.0731**	**0.1229**	**0.1294**	**0.0482**	0.0285	**0.0686**	0.0040		**0.0926**	**0.0467**	0.0433
**CE**	**0.0606**	**0.1072**	**0.0747**	0.0134	0.0217	**0.0455**	0.0342	**0.0575**		0.0198	0.0017
**CU**	**0.0823**	**0.1416**	**0.1171**	0.0307	0.0416	0.0131	0.0247	0.0278	0.0163		−0.0024
**BA**	**0.0531**	**0.1144**	**0.0948**	0.0212	0.0202	**0.0448**	0.0121	0.0280	−0.0001	−0.0009	

*D* values are shown above the diagonal and F_ST_ values below the diagonal. In **bold** are significant comparisons after sequential Bonferroni correction. Population abbreviation names as in Table 1.

### Spatial ordination of the studied populations

**Figure 2:**
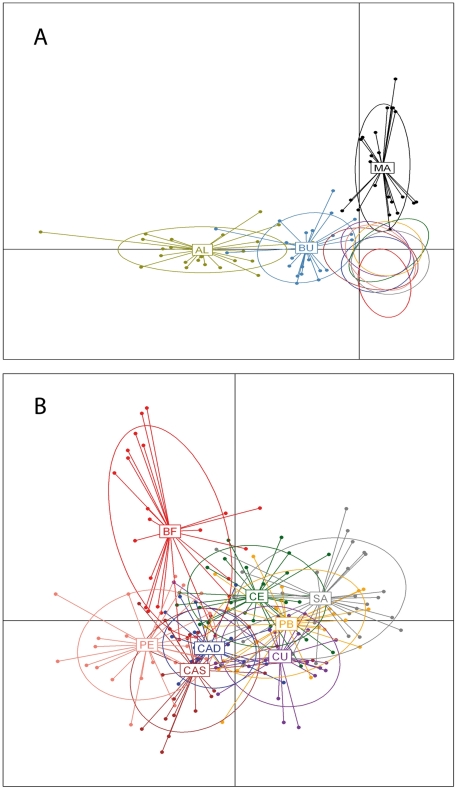
Plots of the first two axes obtained in the Discriminant Analysis of Principal Components using a combined dataset of microsatellite and mtDNA data. Labels were placed at the centre of dispersion of each group, further delineated by inertia ellipses. Dots represent individuals. A) Plot that included all populations. For the introduced populations, we only included ellipses to avoid cluttering, B) Plot including data of only the introduced populations, using the same colour codes as in (A). Population names abbreviated as in Table 1.

The DAPC showed that the 50 principal components of the retained PCA explained 95.3% of the total variance. The scatterplot of the first two components of the DA (Fig. 2A) showed that the native populations were set apart from the introduced ones, which formed a tight cluster with no discernible structure. The first axis separated the two Australian sites of Albany and Bunbury populations from the rest, while the second axis set apart the other Australian population (Manly). No clear overlap of the inertia ellipses existed between the introduced and the native populations, although Bunbury and Manly appeared closer to the introduced group than Albany. When we considered individuals belonging to four groups (three for each of the Australian populations and a single group for the introduced ones), a high proportion (92.2%) of individuals were correctly assigned to their original group using the classification functions obtained in the DA. We then repeated the analysis using only the introduced populations to infer subtle patterns that could have been obscured by the analysis of the entire dataset. A sum of 98.5% of the total variance was then explained by the 50 retained principal components of the PCA. The populations appeared mixed in the space delimited by the first two axes of the DA (Fig. 2B). The first axis showed the South Africa population at one extreme and Santander (Spain) at the other. The second axis mainly separated Bahía Falsa (California) from the rest. The percentage of individuals correctly assigned to their population of origin was relatively low (67.3%), which was in accordance with the low population differentiation observed.

**Figure 3:**
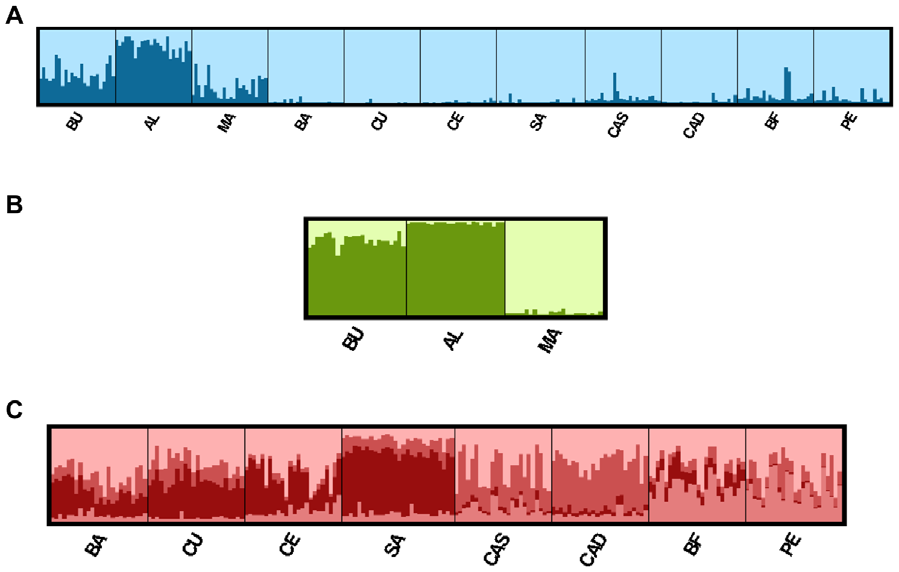
Population structure in native and introduced populations of *Microcosmus squamiger* with the most likely number of populations (K) inferred with the STRUCTURE programme. A) Including all populations, B) Native populations, and C) Introduced populations. Population abbreviations as in Table 1.

When we ran the analysis using the programme STRUCTURE and included all populations, IncK presented the highest value for K = 2 (Fig. S2A), suggesting the presence of two genetically differentiated units. The graphical representation of all populations using K = 2 (Fig. 3A) revealed that the Australian population of Albany had a higher percentage of individuals assigned to one of the two groups, while the introduced populations included the majority of individuals belonging to the other group with a probability of assignment higher than 80%. The individuals belonging to the other two Australian populations (Manly and Bunbury) were mostly assigned to the same group of the introduced populations although the probability of assignment was much lower (Fig. 3A). When we repeated the analysis with only the native populations, IncK was the highest for K = 2 (Fig. S2B) and the graphical output grouped individuals from the western (Bunbury) and southwestern (Albany) Australian populations which appeared highly differentiated from Manly, the eastern population (Fig. 3B). In the analysis including only introduced populations, the highest IncK was observed for K = 4 (Fig. S2C). The resulting assignment detected mixed origin for all individuals with varying contributions from the four clusters (Fig. 3C). However, four groups were observed: the first included individuals from South Africa and California (Port Elizabeth and Bahía Falsa, respectively), the second consisted of a single population (Santander), which was the most differentiated of the four groups (Table 2), the third group included the other Atlantic populations (Cascais and Cádiz), while the fourth group consisted of all Mediterranean populations (Barcelona, Cubelles and Ceuta).

### Revealing the colonisation histories and invasion routes of *M. squamiger* using ABC methods

**Figure 4:**
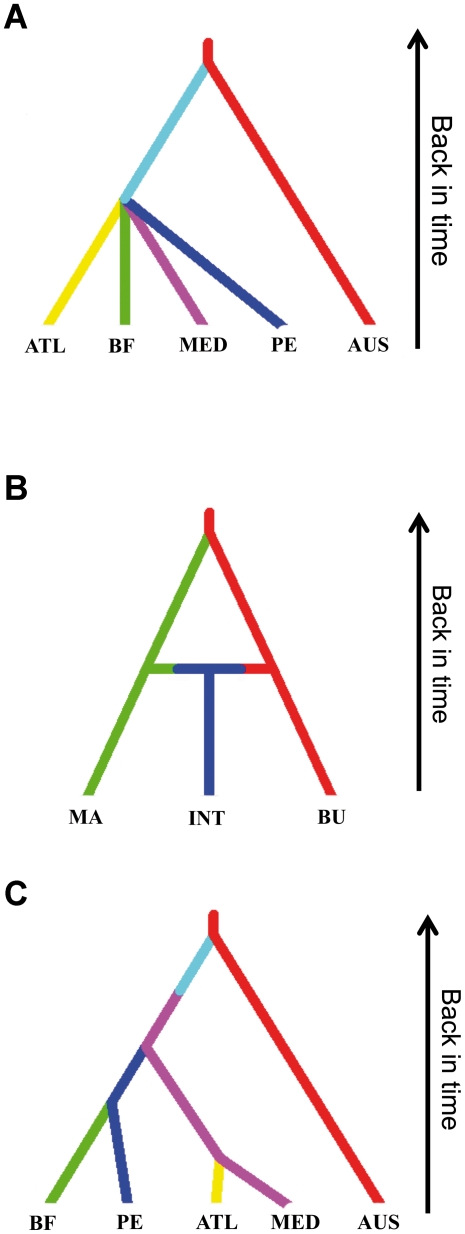
Most-likely scenarios of each set of scenarios using approximate Bayesian computation methods on microsatellite data of *Microcosmus squamiger*: A) Independent vs non-independent colonisations; B) Origin of colonising populations, indicating the admixture process between the two ancestral populations; C) Sequence of worldwide introductions. The Y-axis indicates the time of events (not to scale). Abbreviations of single and clustered populations are as follows: Bahía Falsa (BF), Bunbury (BU), Manly (MA), Port Elizabeth (PE), Introduced (INT), Australian (AUS), NE Atlantic (ATL) and Mediterranean Sea (MED). The unsampled population in A) and C) is indicated by a faint blue colour.

We found a good fit between the scenario-posterior combination and the pseudo-observed data for all sets of scenarios, as shown by both the PCAs and the comparisons between the observed and simulated summary statistics (i.e. model-check option) as implemented in the DIYABC programme (data not shown). The results from the first set of scenarios convincingly showed that the most likely scenario was the one that included non-independent colonisations across *M. squamiger*'s introduced range (Table 3, Fig. 4A). When testing the reputed origin of the colonisers (i.e. the second set of scenarios), the results favoured the admixed scenario when the analysis was run only with microsatellites (Table 3, Fig. 4B). However, when the analysis was run with microsatellite and mtDNA data together, the most likely scenario was the one that assumed Bunbury as the single origin of the introduced populations (Table 3). To resolve these conflicting results, we ran an analysis with a dataset that only included mtDNA and we obtained contradictory results between the direct and logistic regression estimates of the posterior probabilities - the direct estimate showed that the most likely scenario was the one that considered Manly (eastern Australia) as the only origin of colonisers (P = 0.926, C.I = 0.696, 1.000), while the logistic regression showed that the most supported scenario was the one that considers Bunbury (western Australia) as the origin (P = 0.978, C.I. = 0.867, 1.000). Thus, the outcome of the direct estimate was in accordance with a previous study [33] but the logistic regression supported a different outcome. Considering this lack of consistency of the posterior probability, we considered that the DIYABC programme could be selecting incorrect scenarios when divergent population histories revealed by mtDNA and nuclear DNA were mixed, possibly due to selection acting upon mtDNA. Therefore, we considered the results obtained with unlinked microsatellite loci as the most plausible.

**Table 3:** Posterior probabilities and 95% confidence interval of the competing scenarios of each set of scenarios using approximate Bayesian computation methods.

Set of Scenarios	Scenario	Dataset	Posterior Prob	Confidence Interval
*Independent vs non-independent colonisations*				
AUS→PE; AUS→MED; AUS→BF; AUS→ATL	1	MICROS	0.0000	[0.0000,0.0000]
AUS→MED; AUS→PE; AUS→BF; AUS→ATL	2	MICROS	0.0000	[0.0000,0.0000]
**AUS→U→Merge four introduced regions**	**3**	**MICROS**	**1.0000**	**[1.0000,1.0000]**
AUS→PE; AUS→MED; AUS→BF; AUS→ATL	1	MICROS+mtDNA	0.0000	[0.0000,0.0000]
AUS→MED; AUS→PE; AUS→BF; AUS→ATL	2	MICROS+mtDNA	0.0000	[0.0000,0.0000]
**AUS→U→Merge four introduced regions**	**3**	**MICROS+mtDNA**	**1.0000**	**[1.0000,1.0000]**
*Origin of colonising populations*				
BU→MA; MA→Pooled introduced populations	1	MICROS	0,0004	[0.0002,0.0005]
BU→MA; BU→Pooled introduced populations	2	MICROS	0.3448	[0.2892,0.4003]
**BU→MA; BU+MA→Pooled introduced populations**	**3**	**MICROS**	**0.6549**	**[0.5994,0.7104]**
BU→MA; MA→Pooled introduced populations	1	MICROS+mtDNA	0.0048	[0.0000,0.0104]
**BU→MA; BU→Pooled introduced populations**	**2**	**MICROS+mtDNA**	**0.8241**	**[0.6654,0.9829]**
BU→MA; BU+MA→Pooled introduced populations	3	MICROS+mtDNA	0.1710	[0.0166,0.3254]
*Sequence of worldwide introduction*				
AUSc→U; U→MED; U→PE; U→BF; U→ATL	1	MICROS	0.0079	[0.0058,0.0099]
AUSc→U; U→PE; U→MED; U→BF; U→ATL	2	MICROS	0.0305	[0.0232,0.0378]
AUSc→U; U→MED; U→PE; U→BF; MED→ATL	3	MICROS	0.1980	[0.1660,0.2300]
AUSc→U; U→PE; U→MED; U→BF; MED→ATL	4	MICROS	0.1971	[0.1629,0.2313]
**AUSc→U; U→MED; MED→PE; PE→BF; MED→ATL**	**5**	**MICROS**	**0.2710**	**[0.2292,0.3128]**
AUSc→U; U→PE; PE→MED; PE→BF; MED→ATL	6	MICROS	0.1666	[0.1368,0.1965]
AUSc→U; U→PE; PE→ATL; ATL→MED; PE→BF	7	MICROS	0.1015	[0.0804,0.1227]
AUSc→U; U→BF; BF→PE; PE→ATL; ATL→MED	8	MICROS	0.0274	[0.0207,0.0340]
AUSc→U; U→MED; U→PE; U→BF; U→ATL	1	MICROS+mtDNA	0.0122	[0.0071,0.0173]
AUSc→U; U→PE; U→MED; U→BF; U→ATL	2	MICROS+mtDNA	0.0300	[0.0186,0.0415]
**AUSc→U; U→MED; U→PE; U→BF; MED→ATL**	**3**	**MICROS+mtDNA**	**0.2889**	**[0.2198,0.3579]**
AUSc→U; U→PE; U→MED; U→BF; MED→ATL	4	MICROS+mtDNA	0.2210	[0.1613,0.2806]
AUSc→U; U→MED; MED→PE; PE→BF; MED→ATL	5	MICROS+mtDNA	0.1664	[0.1200,0.2129]
AUSc→U; U→PE; PE→MED; PE→BF; MED→ATL	6	MICROS+mtDNA	0.1243	[0.0881,0.1605]
AUSc→U; U→PE; PE→ATL; ATL→MED; PE→BF	7	MICROS+mtDNA	0.1273	[0.0891,0.1656]
AUSc→U; U→BF; BF→PE; PE→ATL; ATL→MED	8	MICROS+mtDNA	0.0298	[0.0199,0.0398]

The dataset used (microsatellite loci - MICROS or both MICROS and mtDNA) is indicated. The scenarios with the highest probability are shown in **bold**. Abbreviations of single and clustered populations are as in Figure 4. ‘U’ indicates the unsampled population. Scenario numbers are as in Figure S2.

Regarding the third set of scenarios (i.e. sequence of worldwide introductions), no scenario could be conclusively selected because the confidence intervals of the scenarios with the highest posterior probabilities overlapped with the intervals from other scenarios (Table 3). However, when considering the microsatellite data alone, the confidence intervals of the scenario with the highest probability (scenario 5, Fig. 4C) marginally overlapped with those of scenarios 3 and 4 (Table 3). Scenario 5 represented a sequential colonisation of the introduced range from an unsampled population that was in accordance with the historical taxonomic records. Conversely, the highest probability for the dataset including both microsatellites and mtDNA was scenario 3 (Table 3), although its confidence intervals overlapped with those of scenario 4. For the same reasons stated above we considered the results obtained with unlinked microsatellite loci as the most reliable.

The demographic parameters obtained for the most supported scenario (i.e. the scenario with the highest posterior probability) of each set of scenarios are shown in Table S5. A high effective population size was detected in both native and introduced populations, although this was slightly larger in the former. The colonisation times among the sites of the introduced range differed by approximately 100 years. When we tested, for the first set of scenarios, the robustness of our inferences on demographic parameters using different priors, the posterior probability indicated that under all prior sets the most supported scenario was Scenario 3 (Table S6, Fig. S1A). Regarding the inferences on demographic parameters, the estimated values of colonization times varied slightly among prior sets, while the differences of effective population sizes were not as negligible (Table S6). Nevertheless, we found that in all cases the confidence intervals for the posterior probabilities were the smallest for the standard prior (prior set 1), which was used to test the different sets of scenarios.

## Discussion

### Genetic diversity and differentiation

The results of the present study are consistent with a scenario of high population connectivity among introduced populations and absence of genetic bottleneck. The analysis of microsatellite data of *M. squamiger* showed that the introduced populations have elevated levels of genetic diversity, similar to those found in native populations. The role of genetic diversity during the colonisation process has been at the heart of recent debates in invasion biology research [15], [90]–[93]. This is because the relative importance of the trade-off between loses of genetic diversity due to bottleneck processes during colonisation, and high genetic diversity as a result of multiple origins or introduction events, remains to be fully understood. The results found for *M. squamiger* indicated that the spread of introduced populations has not been limited by reductions of genetic diversity both in terms of nuclear (this study) and mtDNA [33]. However, we found higher allelic richness and a higher number of private alleles in native populations compared to the introduced ones, as previously seen for mtDNA [33], although these differences were not large. This indicates that despite an absence of bottlenecks in the introduced populations, part of the genetic make-up of the species was lost during the colonisation process and only retained in the native area.

Strong genetic differentiation was detected among Australian populations using microsatellite loci, with a major genetic break between the population found on the eastern coast and the two western populations. This genetic differentiation was observed by pairwise comparisons, either using *D* or F_ST_ values, as well as with STRUCTURE and DAPC. Moreover, this genetic barrier was also found using F_ST_ statistics on mtDNA data from the same populations [33]. This genetic break could be due to isolation by distance with low connectivity between these two coastal regions or as a result of selection due to local adaptation [94]. However, caution is necessary to interpret these findings, as we only had data from three Australian populations and thus we had a limited representation of the genetic diversity in that region. Further studies with an increased sampling size within the native range of *M. squamiger* are needed to ensure a fine-scale analysis of this pattern.

The introduced populations had smaller values of differentiation (both estimated as *D* and as F_ST_) than those found when comparing native populations. Only 43% (*D*) and 29% (F_ST_) of the pairwise comparisons among introduced populations showed significant differentiation in terms of allelic frequencies. A higher genetic similarity among introduced populations than among native ones was also shown by the clustering of these populations in the DAPC and STRUCTURE. This similarity among introduced populations was also reflected by the presence of some loci of low frequency alleles that were only shared among introduced populations (see Table S2). Finally, ABC methods supported non-independent colonisation in distant introduced populations, suggesting recurrent human-mediated transportation among basins, as observed in other invasive species [15], [26], [90], [95]. When we tested for isolation by distance by correlating genetic and geographic distances between all population pairs using a Mantel test (data not shown), we found marginally significant correlations among genetic divergence and geographical distance. When this analysis was restricted to the introduced populations, we found a non-significant relationship. The lack of correlation between genetic and geographic distance among introduced populations strongly points to the role of anthropogenic dispersal as a key factor shaping the genetic composition of these introduced populations. Therefore, transoceanic ship transport has enabled the colonisation of localities that are separated by long distances.

### Tracking the origin of introduced populations

In the present study, the Australian sites of Bunbury and Manly comprised the native populations that appeared closer to the introduced populations in the DAPC and STRUCTURE. In line with this, the ABC analyses supported an admixture of both Manly and Bunbury as a source of the introduced populations, when the analysis was run with microsatellite loci only. However, when both mtDNA and microsatellite markers were combined the most likely scenario that emerged was the one considering Bunbury as the origin of colonisers. This contradictory result might be indicative of different evolutionary processes affecting nuclear and mitochondrial markers [96]. Mitochondrial DNA acts as a single unit due to the lack of recombination, thus selection in one mtDNA locus might leave a footprint in all remaining mitochondrial loci by hitchhiking. In line with this, it has been shown that this widely used marker is affected by selection in animals, which questions its usefulness in evolutionary studies [84]. However, we must also consider the possibility that putative source populations remain unsampled.

The results of our study suggest that the widespread distribution of *M. squamiger* has stemmed from the combined introductions of individuals from the east and west coasts of Australia - two highly differentiated areas in terms of genetic composition - through an unsampled bridgehead population. This process might have granted genetic variability and adaptation potential, which could subsequently have promoted the invasion fitness of this species (e.g. [97], [98]). Multiple introductions have been attributed as the driver that enables the establishment of several invasive terrestrial and aquatic species (e.g. [58], [99]) and recurrent human-mediated introductions might promote their range expansion (e.g. [100]). It is remarkable that most of the regions where *M. squamiger* has been introduced are coastal areas with a Mediterranean climate, where this species exhibits phenological cycles that peak in summer [59]. Temperature therefore seems to be an important factor influencing this species' welfare within the introduced areas. It might also represent a relevant factor influencing the distribution of this species worldwide, by limiting the range expansion of *M. squamiger* in other non-Mediterranean climatic regions, especially those at high latitudes.

### Tracking the sequence of introduction

The ABC analyses based on microsatellite data revealed a sequence that matched with the temporal series from the historical taxonomic records. This result was surprising considering the extensive artificial transportation of exotic ascidians in recent times (e.g. [35], [49]), which have been responsible of homogenizing the genetic composition among regions, as seen in other widespread species [101], [102].

We found a strong link between the populations of the NE Atlantic Ocean and the Mediterranean Sea, while California and South Africa appeared as genetically separated regions. The close genetic relationship between the NE Atlantic Ocean populations and those from the Mediterranean Sea could be a matter of geographical proximity that enhanced connectivity both through commercial and recreational shipping [42], [103] within the region. The Atlanto-Mediterranean transition offers a unique geographic location for the study of marine biological invasions as it has a high concentration of harbours and marinas in the area, and shipping has been intense for many centuries. Genetic studies analysing the similarity between populations of benthic organisms of this region indicate that the Strait of Gibraltar represents an important barrier for dispersal (see [104]–[106]), although human mediated transport may easily overcome this obstacle [107], [108]. In the case of *M. squamiger*, Rius et al. [33] indicated that the Strait of Gibraltar did not seem to act as an important barrier for this species. The results of the present study, however, do not seem to support this hypothesis, as three groups based on STRUCTURE were detected. The first group comprised the Mediterranean populations (Ceuta, Barcelona, Cubelles), which had a similar genetic composition and a consequent lack of significant differences in allele frequencies. The high genetic similarity between these populations could be due to a higher rate of human transportation within the same basin. The second group included two of the Atlantic populations, namely Cascais and Cadiz, and their similarity could be as a result of shipping. The last group comprised the Atlantic population of Santander, which appeared highly differentiated from the rest of the populations. This population also appeared in a peripheral position in the DAPC. Given that this introduced population also had higher allelic richness than the others in the area, we hypothesize that this population might have been established before the other NE Atlantic populations by a larger number of individuals or that recurrent introductions might have shaped its genetic composition. If Santander was excluded, the genetic differentiation (*D* values) in inter-basin (i.e. on both sides of the Strait of Gibraltar) comparisons were low (0.048±0.010, mean ± SE), but much higher than those in intra-basin comparisons (0.006±0.005), which indicates that the Strait of Gibraltar (and associated intense shipping) plays a relevant role in structuring these populations.

In the present study we revealed important characteristics of the introduction process of a widespread marine species. Firstly, we obtained consistent evidence of the non-independent nature of the world colonisations. Subsequently, we found that an admixture event between populations from the east and west Australian coasts was most likely responsible for shaping the genetic composition of the introduced populations of the species. In addition, this admixture event resulted on an unsampled population that served as a stepping-stone between native and introduced populations. Finally, we found that the sequence for the introduced range followed the historical taxonomic records, with the first colonisations occurring in the Mediterranean Sea and South Africa, followed by California and thereafter in the NE Atlantic.

We showed that the combined use of multilocus data obtained from samples of both native and introduced populations with new analytical methods allows for discrimination among complex evolutionary scenarios, and is a powerful approach to understand invasion histories of widespread marine organisms. The translocation of coastal marine species from one distant region to another is increasing [57], [65], [109], [110], enhancing admixture processes and invasive bridgehead effects, which ultimately facilitate marine introduced species to spread and thrive within their introduced range. Such processes enhance NIS population connectivity and constitute an increased threat to native communities.

## Supporting Information

Figure S1 **Set of scenarios used to infer the colonisation histories of** ***Microcosmus squamiger*** **using approximate Bayesian computation analyses**: A) Independent vs non-independent colonisations, B) Origin of colonising populations, C) Sequence of worldwide introductions. The Y-axis indicates the prior time of events (not to scale) as in Table S1. Abbreviations for populations and groups of populations are as in Figure 4. The unsampled population in A) and C) is indicated by a faint blue colour, and the temporal parameters are as in Table S1.
(DOC)

Figure S2 **Plot of IncK as described in Evanno et al.** [**80**] **as a function of the number of clusters (K) across the 20 runs**: (A) for the whole dataset, (B) for the native populations and (C) for the introduced populations.
(DOC)

Table S1 **Prior distribution parameters describing the set of scenarios investigated for** ***Microcosmus squamiger*** **.** The time priors were constrained (t1<t2<t3<t4<t5<t6) and comprised of split or admixed events. Nau: effective population size of the ancestral populations, Nu: effective population size of the unsampled bridgehead population, N: effective population size of each cluster of introduced populations, SNI: single nucleotide indel. All populations had the same prior regarding their effective population size.
(DOC)

Table S2 **List of allele frequencies found in each population of** ***Microcosmus squamiger*** **.** Population abbreviations as in Table 1.
(DOC)

Table S3 **Inbreeding coefficient (F_IS_) and its significance (significant results in bold) for each locus in each population of** ***Microcosmus squamiger*** **.** Total number of alleles (N_A_) per locus are indicated.
(DOC)

Table S4 **Probability of Wilcoxon signed rank test to detect bottlenecks for the introduced populations of** ***Microcosmus squamiger*** **.** Mutation model abbreviations are as follows: IAM - Infinite alleles model and TPM - Two-phase model.
(DOC)

Table S5 **Demographic parameters obtained for the most supported scenario in each set of scenarios using microsatellite markers (see** **Table 3** **).** Demographic parameter abbreviations as in Table S1. The establishment of the unsampled population (t5), colonisation of the Mediterranean Sea (t4), Southern Africa (t3), North America (t2) and NE Atlantic (t1) regions, are indicated.
(DOC)

Table S6 **Robustness of prior choice in discriminating among introduction scenarios (** **Table 3** **, Independent** ***vs*** **non-independent colonisations) and inferences of demographical parameters.** All values correspond to the highest probability scenario in each prior set (i.e. Scenario 3).
(DOC)
