# Supplementary figures and images for: Tracking Invasion Histories in the Sea: Facing Complex Scenarios Using Multilocus Data
Source: PLoS One. 2012 Apr 24;7(4):e35815. doi: 10.1371/journal.pone.0035815 (PMC3335797; doi:10.1371/journal.pone.0035815)

**Figure S1**.

A)


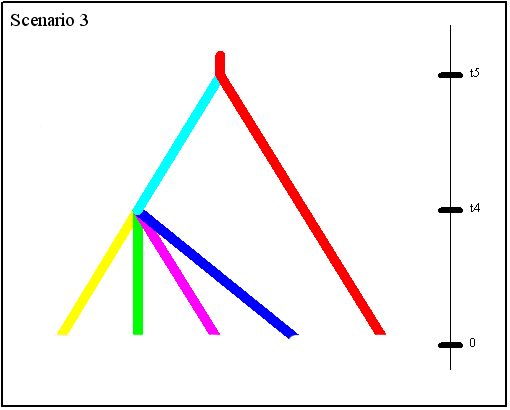


**ATL**

**PE**

**AUS**

**MED**

**BF**


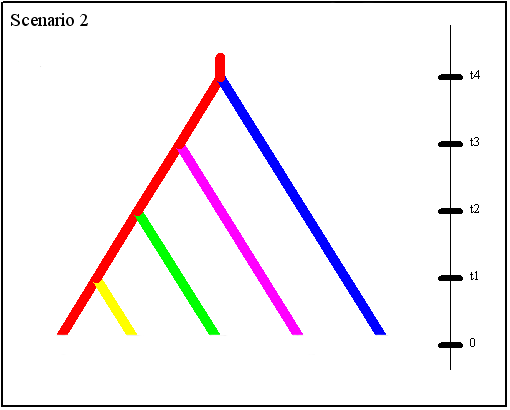


**AUS**

**MED**

**BF**

**PE**

**ATL**


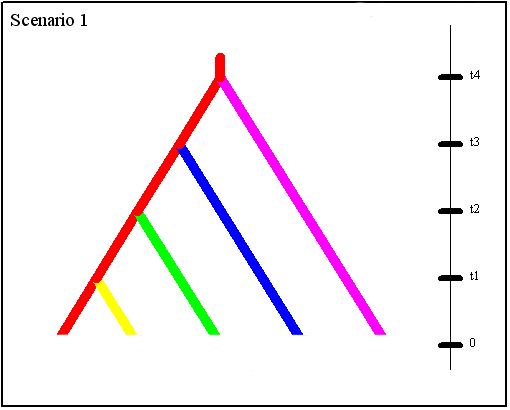


**AUS**

**ATL**

**PE**

**BF**

**MED**

B)


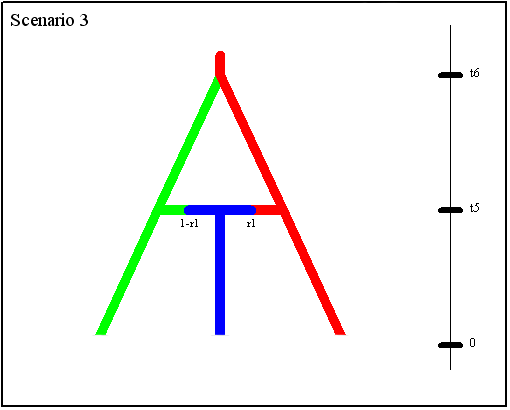


**INT**

**MA**

**BU**


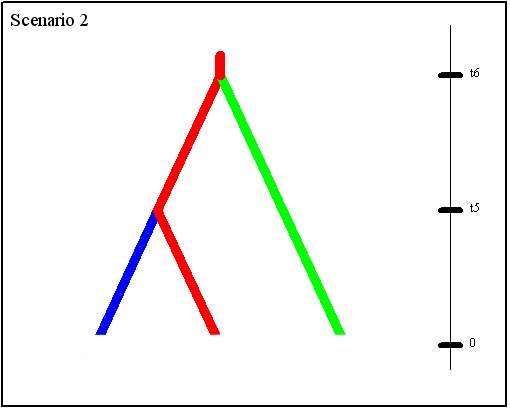


**INT**

**MA**

**BU**


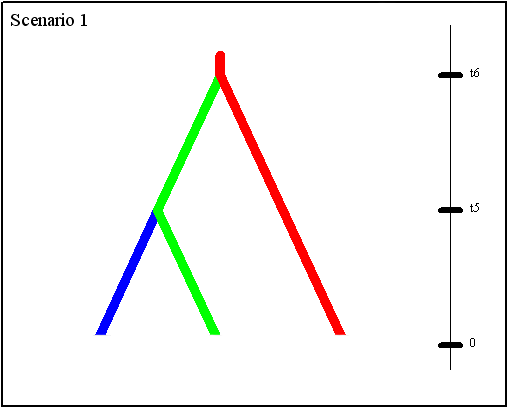


**INT**

**MA**

**BU**

C)

Supplement: Figure S1 — Set of scenarios used to infer the colonisation histories of Microcosmus squamiger using approximate Bayesian computation analyses: A) Independent vs non-independent colonisations, B) Origin of colonising populations, C) Sequence of worldwide introductions. The Y-axis indicates the prior time of events (not to scale) as in Table S1. Abbreviations for populations and groups of populations are as in Figure 4. The unsampled population in A) and C) is indicated by a faint blue colour, and the temporal parameters are as in Table S1. (DOC) [file pone.0035815.s001.doc]
