# Supplementary material for: Tracking Invasion Histories in the Sea: Facing Complex Scenarios Using Multilocus Data
Source: PLoS One. 2012 Apr 24;7(4):e35815. doi: 10.1371/journal.pone.0035815 (PMC3335797; doi:10.1371/journal.pone.0035815)
Supplement: Figure S2 — Plot of IncK as described in Evanno et al. [80] as a function of the number of clusters (K) across the 20 runs: (A) for the whole dataset, (B) for the native populations and (C) for the introduced populations. (DOC) [file pone.0035815.s002.doc]

**Figure S2**.

A)

B)

C)
