# Supplementary material for: Tracking Invasion Histories in the Sea: Facing Complex Scenarios Using Multilocus Data
Source: PLoS One. 2012 Apr 24;7(4):e35815. doi: 10.1371/journal.pone.0035815 (PMC3335797; doi:10.1371/journal.pone.0035815)
Supplement: Table S1 — Prior distribution parameters describing the set of scenarios investigated for Microcosmus squamiger . The time priors were constrained (t1<t2<t3<t4<t5<t6) and comprised of split or admixed events. Nau: effective population size of the ancestral populations, Nu: effective population size of the unsampled bridgehead population, N: effective population size of each cluster of introduced populations, SNI: single nucleotide indel. All populations had the same prior regarding their effective population size. (DOC) [file pone.0035815.s003.doc]

Table S1

| **Parameter** | **Distribution** | **Min.** | **Max.** |
| --- | --- | --- | --- |
| *Effective population size* |  |  |  |
| Nau, N, Nu | Uniform | 10 | 100000 |
| *Time of events (in generations backward in time)* |  |  |  |
| t1, t2, t3, t4 | Uniform | 1 | 200 |
| t5 | Uniform | 100 | 800 |
| t6 | Uniform | 2000 | 20000 |
| *Admixture rate* |  |  |  |
| r1 | Uniform | 0.001 | 0.999 |
| **Microsatellites ^1^** |  |  |  |
| *Mean mutation rate* | Uniform | 1x10^-7^ | 1x10^-4^ |
| *Mean SNI rate* | Log-U | 1x10^-9^ | 1x10^-3^ |
| **COI ^2^** |  |  |  |
| *Mean mutation rate* | Uniform | 1x10^-9^ | 1x10^-6^ |

*Footnote*

**^1^** *Summary statistics included*: mean number of alleles, expected heterozygosity and allele size variance per population, and F_ST_ between population pairs.

**^2^** *Summary statistics included*: number of distinct haplotypes, number of segregating sites, mean pairwise differences, variance of the number of pairwise differences and F_ST_ between population pairs.
