# Supplementary material for: Tracking Invasion Histories in the Sea: Facing Complex Scenarios Using Multilocus Data
Source: PLoS One. 2012 Apr 24;7(4):e35815. doi: 10.1371/journal.pone.0035815 (PMC3335797; doi:10.1371/journal.pone.0035815)
Supplement: Table S2 — List of allele frequencies found in each population of Microcosmus squamiger . Population abbreviations as in Table 1. (DOC) [file pone.0035815.s004.doc]

**Table S2**

|  |  | **Populations** | | | | | | | | | | |
| --- | --- | --- | --- | --- | --- | --- | --- | --- | --- | --- | --- | --- |
|  |  | **BU** | **AL** | **MA** | **BF** | **PE** | **SA** | **CAS** | **CAD** | **CE** | **CU** | **BA** |
| **LOCI** | **Allele** |  |  |  |  |  |  |  |  |  |  |  |
| **MS6** |  |  |  |  |  |  |  |  |  |  |  |  |
|  | 149 | 0.146 | 0.438 | 0.104 | 0.229 | 0.333 | 0.018 | 0.146 | 0.229 | 0.188 | 0.042 | 0.125 |
|  | 165 | 0.063 | 0.083 | 0.021 | 0.042 | 0 | 0.143 | 0.083 | 0.042 | 0.042 | 0.146 | 0.083 |
|  | 200 | 0.792 | 0.458 | 0.729 | 0.667 | 0.667 | 0.357 | 0.771 | 0.688 | 0.563 | 0.563 | 0.625 |
|  | 205 | 0 | 0 | 0.125 | 0.063 | 0 | 0.482 | 0 | 0 | 0.167 | 0.25 | 0.167 |
|  | 207 | 0 | 0 | 0 | 0 | 0 | 0 | 0 | 0 | 0.042 | 0 | 0 |
|  | 218 | 0 | 0 | 0.021 | 0 | 0 | 0 | 0 | 0 | 0 | 0 | 0 |
|  | 252 | 0 | 0.021 | 0 | 0 | 0 | 0 | 0 | 0.042 | 0 | 0 | 0 |
| **MS7** |  |  |  |  |  |  |  |  |  |  |  |  |
|  | 296 | 0.021 | 0 | 0 | 0 | 0 | 0 | 0 | 0 | 0 | 0 | 0.021 |
|  | 306 | 0.063 | 0.125 | 0.021 | 0.042 | 0 | 0.054 | 0 | 0 | 0.042 | 0 | 0 |
|  | 312 | 0.854 | 0.75 | 0.729 | 0.854 | 0.792 | 0.643 | 0.792 | 0.646 | 0.792 | 0.708 | 0.729 |
|  | 315 | 0.063 | 0.125 | 0.25 | 0.104 | 0.208 | 0.304 | 0.208 | 0.354 | 0.167 | 0.292 | 0.25 |
| **MS10** |  |  |  |  |  |  |  |  |  |  |  |  |
|  | 307 | 0 | 0 | 0.042 | 0 | 0 | 0 | 0 | 0 | 0 | 0 | 0 |
|  | 308 | 0.146 | 0 | 0 | 0.205 | 0.167 | 0.262 | 0.083 | 0.159 | 0.1 | 0.026 | 0.063 |
|  | 309 | 0 | 0 | 0.042 | 0 | 0 | 0 | 0 | 0 | 0 | 0 | 0 |
|  | 311 | 0.021 | 0 | 0.021 | 0 | 0 | 0 | 0.021 | 0 | 0.025 | 0 | 0 |
|  | 312 | 0.021 | 0.026 | 0 | 0 | 0 | 0 | 0 | 0 | 0 | 0 | 0 |
|  | 328 | 0 | 0 | 0 | 0 | 0 | 0 | 0 | 0 | 0 | 0 | 0.021 |
|  | 349 | 0.146 | 0.632 | 0.021 | 0 | 0.021 | 0.024 | 0.042 | 0 | 0 | 0 | 0.021 |
|  | 350 | 0 | 0 | 0 | 0 | 0 | 0 | 0 | 0 | 0 | 0.053 | 0 |
|  | 359 | 0 | 0 | 0.042 | 0 | 0 | 0 | 0 | 0 | 0 | 0 | 0 |
|  | 361 | 0.5 | 0.237 | 0.813 | 0.682 | 0.708 | 0.619 | 0.729 | 0.818 | 0.8 | 0.789 | 0.875 |
|  | 378 | 0.021 | 0.026 | 0 | 0 | 0.042 | 0 | 0 | 0 | 0 | 0 | 0 |
|  | 379 | 0 | 0 | 0 | 0 | 0 | 0 | 0 | 0 | 0 | 0.026 | 0 |
|  | 414 | 0 | 0 | 0 | 0.023 | 0 | 0 | 0 | 0 | 0 | 0 | 0 |
|  | 417 | 0 | 0 | 0.021 | 0 | 0 | 0 | 0 | 0 | 0 | 0 | 0 |
|  | 434 | 0 | 0 | 0 | 0 | 0 | 0.024 | 0 | 0 | 0 | 0.026 | 0 |
|  | 435 | 0 | 0 | 0 | 0 | 0.021 | 0 | 0 | 0 | 0 | 0 | 0 |
|  | 440 | 0.125 | 0.079 | 0 | 0.091 | 0.042 | 0.048 | 0.104 | 0.023 | 0.075 | 0.079 | 0.021 |
|  | 460 | 0.021 | 0 | 0 | 0 | 0 | 0.024 | 0.021 | 0 | 0 | 0 | 0 |
| **MS11** |  |  |  |  |  |  |  |  |  |  |  |  |
|  | 202 | 0.125 | 0.083 | 0.042 | 0.333 | 0.104 | 0.268 | 0.042 | 0.083 | 0.25 | 0.167 | 0.083 |
|  | 204 | 0 | 0 | 0.021 | 0 | 0 | 0 | 0 | 0 | 0 | 0 | 0 |
|  | 207 | 0.208 | 0.146 | 0.292 | 0.313 | 0.313 | 0.268 | 0.271 | 0.188 | 0.438 | 0.458 | 0.438 |
|  | 208 | 0 | 0 | 0 | 0 | 0.188 | 0.036 | 0 | 0 | 0 | 0 | 0 |
|  | 209 | 0.083 | 0.146 | 0.083 | 0.063 | 0.083 | 0.125 | 0.229 | 0.375 | 0.063 | 0.167 | 0.167 |
|  | 211 | 0.146 | 0.021 | 0.396 | 0.146 | 0.25 | 0.071 | 0.292 | 0.188 | 0.104 | 0.125 | 0.125 |
|  | 213 | 0.438 | 0.604 | 0.021 | 0.146 | 0.042 | 0.125 | 0.167 | 0.125 | 0.063 | 0.083 | 0.146 |
|  | 214 | 0 | 0 | 0 | 0 | 0.021 | 0.018 | 0 | 0 | 0 | 0 | 0 |
|  | 215 | 0 | 0 | 0 | 0 | 0 | 0.089 | 0 | 0.042 | 0.021 | 0 | 0.042 |
|  | 217 | 0 | 0 | 0 | 0 | 0 | 0 | 0 | 0 | 0.063 | 0 | 0 |
|  | 219 | 0 | 0 | 0.063 | 0 | 0 | 0 | 0 | 0 | 0 | 0 | 0 |
|  | 222 | 0 | 0 | 0.083 | 0 | 0 | 0 | 0 | 0 | 0 | 0 | 0 |
| **MS12** |  |  |  |  |  |  |  |  |  |  |  |  |
|  | 102 | 0.042 | 0 | 0.104 | 0.021 | 0 | 0.036 | 0 | 0 | 0.104 | 0.083 | 0.104 |
|  | 105 | 0 | 0 | 0 | 0 | 0.042 | 0.036 | 0.042 | 0 | 0 | 0 | 0 |
|  | 107 | 0 | 0 | 0 | 0 | 0 | 0 | 0 | 0 | 0.021 | 0 | 0 |
|  | 108 | 0.188 | 0.292 | 0.333 | 0.25 | 0.104 | 0.089 | 0.063 | 0.042 | 0.042 | 0.042 | 0.083 |
|  | 111 | 0.646 | 0.375 | 0.313 | 0.583 | 0.708 | 0.571 | 0.667 | 0.688 | 0.688 | 0.646 | 0.771 |
|  | 113 | 0 | 0.042 | 0 | 0.021 | 0.104 | 0.089 | 0.083 | 0.146 | 0 | 0.125 | 0 |
|  | 115 | 0.083 | 0.021 | 0.229 | 0.042 | 0 | 0.179 | 0.125 | 0.104 | 0.146 | 0.104 | 0.042 |
|  | 120 | 0 | 0 | 0 | 0.042 | 0.042 | 0 | 0 | 0 | 0 | 0 | 0 |
|  | 128 | 0 | 0.021 | 0 | 0 | 0 | 0 | 0 | 0 | 0 | 0 | 0 |
|  | 135 | 0.042 | 0.167 | 0.021 | 0 | 0 | 0 | 0 | 0.021 | 0 | 0 | 0 |
|  | 156 | 0 | 0.042 | 0 | 0.042 | 0 | 0 | 0.021 | 0 | 0 | 0 | 0 |
|  | 171 | 0 | 0.042 | 0 | 0 | 0 | 0 | 0 | 0 | 0 | 0 | 0 |
| **MS13** |  |  |  |  |  |  |  |  |  |  |  |  |
|  | 220 | 0 | 0 | 0.042 | 0.021 | 0 | 0 | 0 | 0 | 0 | 0 | 0 |
|  | 223 | 0.042 | 0.042 | 0 | 0.104 | 0 | 0 | 0.063 | 0 | 0.083 | 0.063 | 0.125 |
|  | 225 | 0.354 | 0.208 | 0.104 | 0.5 | 0.458 | 0.589 | 0.5 | 0.625 | 0.333 | 0.604 | 0.438 |
|  | 226 | 0 | 0 | 0 | 0 | 0 | 0 | 0 | 0 | 0.021 | 0 | 0 |
|  | 227 | 0.354 | 0.375 | 0.125 | 0.104 | 0.167 | 0.161 | 0.167 | 0.104 | 0.146 | 0.063 | 0.083 |
|  | 228 | 0.146 | 0.292 | 0.688 | 0.271 | 0.375 | 0.232 | 0.229 | 0.25 | 0.417 | 0.25 | 0.333 |
|  | 230 | 0.021 | 0 | 0 | 0 | 0 | 0 | 0 | 0 | 0 | 0 | 0 |
|  | 231 | 0.083 | 0 | 0.042 | 0 | 0 | 0.018 | 0.042 | 0.021 | 0 | 0.021 | 0.021 |
|  | 239 | 0 | 0.063 | 0 | 0 | 0 | 0 | 0 | 0 | 0 | 0 | 0 |
|  | 244 | 0 | 0.021 | 0 | 0 | 0 | 0 | 0 | 0 | 0 | 0 | 0 |
