# Supplementary material for: Tracking Invasion Histories in the Sea: Facing Complex Scenarios Using Multilocus Data
Source: PLoS One. 2012 Apr 24;7(4):e35815. doi: 10.1371/journal.pone.0035815 (PMC3335797; doi:10.1371/journal.pone.0035815)
Supplement: Table S3 — Inbreeding coefficient (FIS) and its significance (significant results in bold) for each locus in each population of Microcosmus squamiger . Total number of alleles (NA) per locus are indicated. (DOC) [file pone.0035815.s005.doc]

**Table S3**

| Population/Locus | **MS6** | **MS7** | **MS10** | **MS11** | **MS12** | **MS13** |
| --- | --- | --- | --- | --- | --- | --- |
| Bunbury | 0.064 | **-0.092** | -0.066 | 0.039 | 0.168 | 0.266 |
| Albany | 0.245 | -0.004 | **-0.348** | 0.307 | 0.065 | **0.555** |
| Manly | -0.018 | 0.096 | 0.146 | 0.230 | **0.388** | 0.431 |
| Bahía Falsa | 0.100 | 0.052 | -0.103 | **0.292** | **0.453** | 0.319 |
| Port Elizabeth | 0.084 | **-0.243** | 0.038 | **0.329** | 0.401 | 0.216 |
| Santander | 0.210 | -0.071 | -0.026 | 0.224 | 0.158 | 0.145 |
| Cascais | 0.028 | 0.011 | -0.002 | **0.306** | **0.695** | **0.636** |
| Cádiz | 0.137 | -0.163 | -0.171 | **0.311** | **0.509** | **0.548** |
| Ceuta | -0.127 | 0.052 | **-0.139** | 0.099 | 0.176 | 0.231 |
| Cubelles | -0.164 | 0.013 | -0.125 | **0.375** | **0.633** | 0.208 |
| Barcelona | 0.274 | **-0.317** | 0.292 | 0.171 | 0.265 | 0.094 |
| N_A_ | 7 | 4 | 18 | 12 | 12 | 10 |
