# Supplementary material for: Tracking Invasion Histories in the Sea: Facing Complex Scenarios Using Multilocus Data
Source: PLoS One. 2012 Apr 24;7(4):e35815. doi: 10.1371/journal.pone.0035815 (PMC3335797; doi:10.1371/journal.pone.0035815)
Supplement: Table S4 — Probability of Wilcoxon signed rank test to detect bottlenecks for the introduced populations of Microcosmus squamiger . Mutation model abbreviations are as follows: IAM - Infinite alleles model and TPM - Two-phase model. (DOC) [file pone.0035815.s006.doc]

**Table S4**

| **Population** | **IAM** | **TPM** |
| --- | --- | --- |
| Bahía Falsa | 0.84 | 0.43 |
| Port Elizabeth | 0.15 | 1 |
| Santander | 0.11 | 0.84 |
| Cascais | 1 | 0.84 |
| Cádiz | 0.56 | 1 |
| Ceuta | 0.84 | 0.11 |
| Cubelles | 0.69 | 1 |
