# Supplementary material for: Tracking Invasion Histories in the Sea: Facing Complex Scenarios Using Multilocus Data
Source: PLoS One. 2012 Apr 24;7(4):e35815. doi: 10.1371/journal.pone.0035815 (PMC3335797; doi:10.1371/journal.pone.0035815)
Supplement: Table S5 — Demographic parameters obtained for the most supported scenario in each set of scenarios using microsatellite markers (see Table 3 ). Demographic parameter abbreviations as in Table S1. The establishment of the unsampled population (t5), colonisation of the Mediterranean Sea (t4), Southern Africa (t3), North America (t2) and NE Atlantic (t1) regions, are indicated. (DOC) [file pone.0035815.s007.doc]

**Table S5**

|  | **Parameter** | **mean** | **mode** | **median** | **Q5%** | **Q95%** |
| --- | --- | --- | --- | --- | --- | --- |
| *Prior* |  |  |  |  |  |  |
|  | Nau, N, Nu | 50415 | 4503 | 52157 | 5223 | 94302 |
|  | t1, t2, t3, t4 | 104 | 195 | 105 | 10 | 194 |
|  | t5 | 446 | 411 | 431 | 138 | 761 |
|  | t6 | 10959 | 2881 | 10924 | 2942 | 18945 |
| *Independent vs non-independent colonisations* | | | | |  |  |
|  | Nau | 26800 | 17900 | 24100 | 11500 | 51800 |
|  | N | 13100 | 2510 | 6240 | 935 | 51500 |
|  | Nu | 16900 | 7190 | 11400 | 2720 | 53900 |
|  | t4 | 171 | 199 | 185 | 89 | 199 |
|  | t5 | 606 | 799 | 631 | 342 | 788 |
| *Origin of colonising populations* | | |  |  |  |  |
|  | Nau | 19400 | 10400 | 15800 | 5980 | 45900 |
|  | N | 46800 | 35400 | 43800 | 15700 | 89300 |
|  | t5 | 665 | 796 | 708 | 383 | 797 |
|  | t6 | 2970 | 2100 | 2430 | 2060 | 5690 |
|  | r1 | 0.62 | 0.637 | 0.637 | 0.34 | 0.85 |
| *Sequence of worldwide introductions* | | | |  |  |  |
|  | Nau | 25300 | 9090 | 19200 | 6030 | 69400 |
|  | N | 5300 | 793 | 1770 | 417 | 23700 |
|  | Nu | 54300 | 33700 | 53700 | 13000 | 95800 |
|  | t1 | 59.8 | 38.7 | 55 | 6.8 | 132 |
|  | t2 | 86.1 | 68.4 | 84.6 | 25.7 | 152 |
|  | t3 | 133 | 159 | 137 | 67.7 | 185 |
|  | t4 | 170 | 199 | 177 | 117 | 199 |
|  | t5 | 507 | 520 | 511 | 225 | 775 |
