# Supplementary material for: Tracking Invasion Histories in the Sea: Facing Complex Scenarios Using Multilocus Data
Source: PLoS One. 2012 Apr 24;7(4):e35815. doi: 10.1371/journal.pone.0035815 (PMC3335797; doi:10.1371/journal.pone.0035815)
Supplement: Table S6 — Robustness of prior choice in discriminating among introduction scenarios ( Table 3 , Independent vs non-independent colonisations) and inferences of demographical parameters. All values correspond to the highest probability scenario in each prior set (i.e. Scenario 3). (DOC) [file pone.0035815.s008.doc]

**Table S6**.

|  |  | **Prior set 1** | **Prior set 2** | **Prior set 3** | **Prior set 4** |
| --- | --- | --- | --- | --- | --- |
|  |  |  |  |  |  |
| Scenario 3 | Probability | 1.0 | 1.0 | 1.0 | 1.0 |
|  |  |  |  |  |  |
| Nau | Mean | 39300 | 64100 | 37300 | 27700 |
|  | Q5% | 19900 | 17100 | 11200 | 11600 |
|  | Q95% | 65900 | 153000 | 83400 | 56200 |
| N | Mean | 11400 | 93700 | 13900 | 8660 |
|  | Q5% | 665 | 2340 | 1090 | 536 |
|  | Q95% | 48100 | 405000 | 53900 | 35900 |
| Nu | Mean | 16500 | 107000 | 18100 | 20300 |
|  | Q5% | 2270 | 3730 | 2690 | 3880 |
|  | Q95% | 53700 | 458000 | 58500 | 60400 |
| t4 | Mean | 184 | 127 | 151 | 147 |
|  | Q5% | 134 | 22.7 | 49.4 | 46.6 |
|  | Q95% | 200 | 196 | 198 | 197 |
| t5 | Mean | 699 | 672 | 637 | 678 |
|  | Q5% | 511 | 437 | 359 | 467 |
|  | Q95% | 794 | 793 | 789 | 793 |

*Footnote*

Prior set 1: standard priors as described in Table S1. Prior set 2: uniform distributions bounded between 10 and 10^6^ diploid individuals for N, Nau and Nu. Prior set 3: stepwise mutation model for microsatellite loci. Prior set 4: insertion-deletion mutation rate in microsatellites flanking regions assumed to be equal to zero.
